# Gas-generated thermal oxidation of a coordination cluster for an anion-doped mesoporous metal oxide

**DOI:** 10.1038/srep18468

**Published:** 2015-12-18

**Authors:** Kenji Hirai, Shigehito Isobe, Kazuki Sada

**Affiliations:** 1 Department of Chemistry, Graduate School of Science, Hokkaido University, Kita 10 Nishi 8, Kita-ku, Sapporo, Hokkaido 060-0810, Japan; 2 Creative Research Institution, Hokkaido University, Kita 21, Nishi 10, Kita-ku, Sapporo, Hokkaido 001-0021, Japan; 3 Graduate School of Engineering, Hokkaido University, Kita 13, Nishi 8, Kita-ku, Sapporo, Hokkaido 060-8628, Japan

## Abstract

Central in material design of metal oxides is the increase of surface area and control of intrinsic electronic and optical properties, because of potential applications for energy storage, photocatalysis and photovoltaics. Here, we disclose a facile method, inspired by geochemical process, which gives rise to mesoporous anion-doped metal oxides. As a model system, we demonstrate that simple calcination of a multinuclear coordination cluster results in synchronic chemical reactions: thermal oxidation of Ti_8_O_10_(4-aminobenzoate)_12_ and generation of gases including amino-group fragments. The gas generation during the thermal oxidation of Ti_8_O_10_(4-aminobenzoate)_12_ creates mesoporosity in TiO_2_. Concurrently, nitrogen atoms contained in the gases are doped into TiO_2_, thus leading to the formation of mesoporous N-doped TiO_2_. The mesoporous N-doped TiO_2_ can be easily synthesized by calcination of the multinuclear coordination cluster, but shows better photocatalytic activity than the one prepared by a conventional sol-gel method. Owing to an intrinsic designability of coordination compounds, this facile synthetic will be applicable to a wide range of metal oxides and anion dopants.

Geochemical process coupled with gas generation is of great importance to the evolution of natural porous minerals. The porosity in the minerals is created by evaporation of gas bubbles. The gases comprised mostly of water steam, carbon dioxide but also contains a small amount of hydrogen sulphide, hydrogen fluoride and ammonia^1^. The anions in those gases react with minerals to be incorporated as anionic partners for metal ions^2^. Consequently, incorporation of anions and void formations in the minerals simultaneously occur, giving rise to natural porous minerals containing anions such as sulphur, fluorine and nitrogen.

Porous metal oxides represent promising materials for energy storage^3^, photocatalysis^4^,^5^, and photovoltaics^6^,^7^ because of the large active surface area. By contrast, the control of chemical composition in metal oxides is also vital to these applications. In particular, incorporation of another anion into metal oxides, i.e. anion doping, provides excellent performance in ion-storage^8^ and photocatalytic reaction^9^,^10^. However, synthesis of porous metal oxides and anion doping have been individually developed. In that context, a crucial challenge in this research field is to coherently integrate these two processes. These considerations inspire us to mimic the geochemical process to establish a facile synthetic method for anion-doped porous metal oxides.

Coordination compounds, wherein metal ions and organic ligands are rationally varied^11^,^12^,^13^,^14^,^15^,^16^, are candidates for precursor to apply the gas-generated thermal oxidation. Indeed, coordination compounds are thermally oxidized into metal oxides by calcination^17^,^18^,^19^,^20^. On the other hand, organic molecules are fragmented to generate gases by intense heating^21^,^22^. In particular, gases containing reactive anions are generated by the fragmentation of organic functional groups, which potentially act as dopant sources. In general, however, the organic ligands of coordination compounds are removed by heating before reaching temperatures where metal oxides are formed. Because of the temperature gap, a calcination of coordination compounds gives metal oxides even without anion doping.

Our strategy to overcome the problem is to improve thermal stability of organic ligands by robust coordination bonding^23^ of carboxylates with a multinuclear metal cluster. As a model system, we design a multinuclear titanium coordination cluster comprised of a carboxylate ligand with a pendant amino-group. The carboxylate ligand is anchored by coordination bonding with the multinuclear titanium cluster until formation of metal oxides. Therefore, fragmentation of amino-group overlaps with thermal oxidation of the titanium coordination cluster. Consequently, TiO_2_ is formed under evaporation of gases containing nitrogen atoms, giving rise to N-doped TiO_2_^24^,^25^,^26^ with permanent porosity. In other words, the porous N-doped TiO_2_ can be obtained by a simple calcination of the coordination cluster.

Metal oxides doped with anion^27^,^28^,^29^ has attracted much attention because of potential applications of visible-light photocatalyst for water splitting^30^, pollutant degradation^31^,^32^ and solar energy conversion^33^,^34^. Porosity further improves the photocatalytic activity by increasing a surface area and improving the accessibility to catalytic active sites^35^. The mesoporous metal oxide has been fabricated by elaborate protocols, including templating method^36^,^37^, or particle assembly^38^,^39^. Sol-gel method is rather simple to synthesize mesoporous metal oxide, which can be easily combined with anion doping^40^,^41^. However, synthesis of mesoporous metal oxides via sol-gel method requires precise control of hydrolysis and condensation rates, which would conflict with anion doping approach. From simplicity of the protocol, calcination of coordination clusters will be an attractive strategy to fabricate anion-doped porous metal oxides (Fig. 1). Notably, coordination compounds can be rationally designed by a judicious choice of metal ions and organic ligands^42^. Therefore, the strategy presented here will be applicable to other types of metal oxides and anion dopants.

## Results

**Figure 2:**
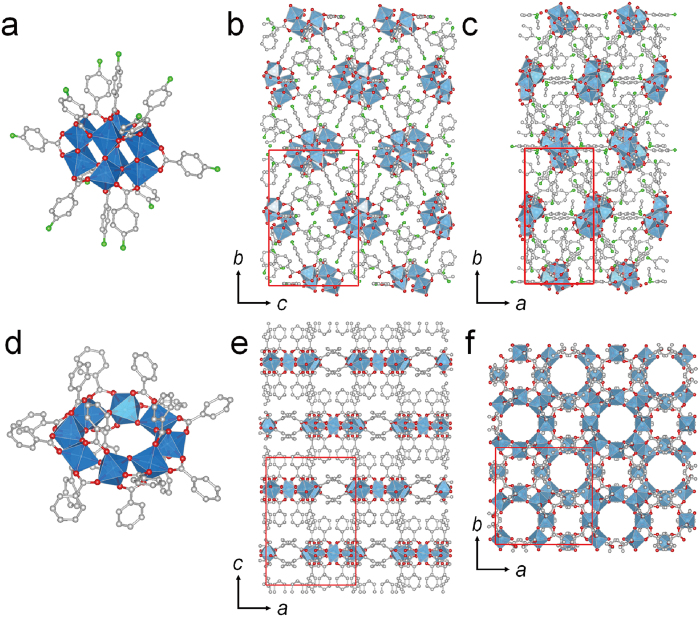
Crystal structures of titanium coordination clusters. Crystal structures of **1** and **2**: (**a**) coordination geometry of **1**. (**b**) view of **1** along *a* axis and (**c**) *c* axis. (**d**) coordination geometry of **2**. (**e**) view of **2** along *b* axis and (**f**) *c* axis. The hydrogen atoms and solvent molecules are omitted for clarity. Each atoms of Ti, oxygen and carbon are coloured by blue, red and white. Ti is shown as a cation centred octahedral geometry.

We synthesized a titanium coordination cluster with 4-amino benzoic acid. A solvothermal reaction of titanium isopropoxide and 4-amino benzoic acid in acetonitrile gave cuboid crystals with a size of several hundred μm. The resulting compound of Ti_8_O_10_(4-aminobenzoate)_12_ (**1**) consists of Ti_8_O_10_ cluster, where octanuclear titanium is linked by ten *μ*_2_-oxo bridges. The carboxyl groups of twelve 4-aminobenzoate further bridge each titanium to each of its neighbouring titanium in a bidentate fashion (Fig. 2a–c).

As a reference, another titanium coordination cluster without amino-group, Ti_8_O_8_(benzoate)_16_^43^ (**2**), was synthesized by a solvothermal reaction of titanium isopropoxide and benzoic acid. The compound (**2**) consists of Ti_8_O_8_ ring-shaped cluster, where octanuclear titanium is linked by eight *μ*_2_-oxo bridges. The carboxyl groups of sixteen benzoate binds to titanium in a bidentate fashion from the axial and equatorial positions (Fig. 2d–f). Eight equatorial benzoate point up and down alternatively from the plane of Ti_8_O_8_ ring cluster, whereas the eight other axial benzoate point up and down perpendicularly.

**Figure 3:**
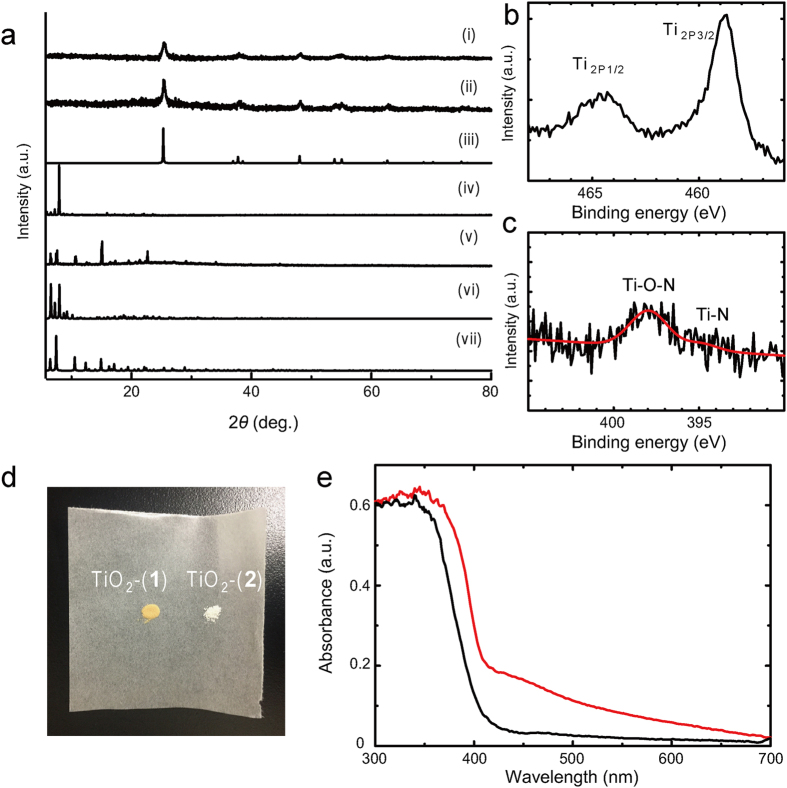
Spectroscopic characterization of TiO_2_-(1) and TiO_2_-(2). (**a**) PXRD of (i) TiO_2_-(**1**), (ii) TiO_2_-(**2**), (iii) Simulated TiO_2_, (iv) **1**, (v) **2**, (vi) simulated **1** and (vii) simulated **2**. (**b–c**) XPS spectra of TiO_2_-(**1**) for Ti_2p_ and N_1s_ with fitting curves (red). The whole spectra is shown in supplementary information. (**d**) Photograph of TiO_2_-(**1**) and TiO_2_-(**2**), (**e**) UV-vis absorption spectra of TiO_2_-(**1**) (red) and TiO_2_-(**2**) (black).

Dozens of crystals of **1** and **2** were calcined at 480 °C in air for 3 hours (heating rate: 8 °C/min). The tiny crystalline particles with the size of 5 μm were obtained by calcination of **1** and **2** (Figure S1). X-ray diffraction (XRD) pattern of calcined **1** and **2** corresponded to anatase TiO_2_, suggesting that **1** and **2** were converted into TiO_2_ (denoted as TiO_2_-(**1**) and TiO_2_-(**2**), respectively) (Fig. 3a).

X-ray photon spectroscopy (XPS) was carried out to clarify the incorporation of nitrogen atoms in TiO_2_. A broad XPS peak of N_1s_ was observed in TiO_2_-(**1**) but not in TiO_2_-(**2**), suggesting that nitrogen in TiO_2_-(**1**) is originating from the amino group of 4-aminobenzoate (Figure S2). The binding energy of N_1s_ (398 eV) corresponded to anionic N^−^ in Ti-O-N which is in the range typically observed for substitutional nitrogen doping into TiO_2_^44^,^45^,^46^. Furthermore, the binding energies of Ti_2p1/2_ (464 eV) and Ti_2p3/2_ (459 eV) well matched with those of Ti in N-doped TiO_2_^47^ (Fig. 3b,c). As shown in Figure S4, Raman spectra of TiO_2_-(**1**) and TiO_2_-(**2**) showed the characteristic blue shift of E_g(1)_ band by nitrogen doping (139.6 cm^−1^ for TiO_2_-(**2**) and 144.0 cm^−1^ for TiO_2_-(**1**))^48^. These results suggested that nitrogen originating from amino group was incorporated into TiO_2_ as a dopant, giving rise to N-doped TiO_2_. The nitrogen concentration in TiO_2_-(**1**) was estimated as 0.96%.

The resulting TiO_2_-(**1**) is yellow because of the nitrogen doping, whereas non-doped TiO_2_, including TiO_2_-(**2**), is white (Fig. 3d). As expected, TiO_2_-(**1**) showed absorption in the visible-light region (400–500 nm), but TiO_2_-(**2**) absorbs only light in ultraviolet (UV) region (Fig. 3e). This is because nitrogen doping into TiO_2_ created a new energy level (N_2p_ level) above the valence band maximum. The new absorption band in 400–450 nm corresponds to the energy gap between conductance band and N_2p_ level (2.7 eV). These results suggest that TiO_2_-(**1**) is able to work as photocatalyst under visible light.

Besides the nitrogen dope into TiO_2_, the porosity of TiO_2_-(**1**) and TiO_2_-(**2**) was evaluated by N_2_ adsorption (Figure S5a). The adsorption/desorption hysteresis was observed for TiO_2_-(**1**) and TiO_2_-(**2**) in the relative pressure (*P*/*P*_0_) range of 0.4–0.9. This characteristic hysteresis is attributed to the mesopores of TiO_2_. The gradual adsorption in the hysteresis region, classified as H2 type adsorption, suggested mesopores with ununiform size and shape. The pore-size distribution, based on the desorption branch of the isotherm, was estimated by Barret, Joyner, and Halender (BJH) method, assuming a cylindrical pore model. The pore sizes of TiO_2_-(**1**) and TiO_2_-(**2**) were calculated to be around 4 nm (Figure S5b). The mesopores of TiO_2_-(**1**) were also observed by TEM (Figure S6). BET surfaces of TiO_2_-(**1**) and TiO_2_-(**2**) were estimated as 170.6 m^2^/g and 139.8 m^2^/g, which were relatively large compared to those of metal oxides prepared by calcination of coordination compounds^49^,^50^.

**Figure 4:**
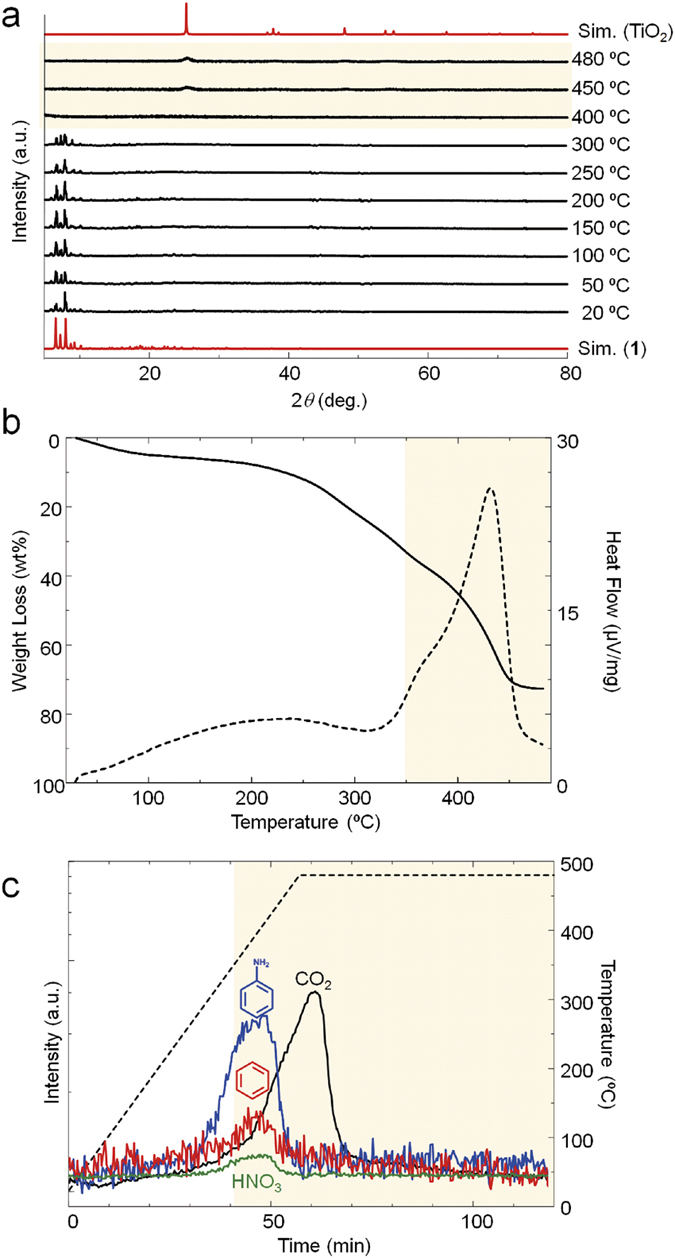
Time course experiments on calcination of the coordination cluster. (**a**) PXRD of **1** at variable temperature 20–480 °C. (**b**) TG analysis showing weight loss of 1 upon heating (black solid). DTA analysis showing exothermal peak around after 350 °C (black dots). (**c**) Q-MS analysis with heating of **1**: aniline (m/z: 95, blue), benzene (m/z: 79, red), CO_2_ (m/z: 44, black) and HNO_3_ (m/z: 63, green) were observed. Black dot line shows temperature of the sample cell. The yellow back ground indicates the temperature region for the formation of TiO_2_.

The series of measurements indicated that simple calcination of the coordination cluster allows the synthesis of mesoporous N-doped TiO_2_. To investigate the formation mechanism of mesoporous N-doped TiO_2_, variable-temperature XRD (VT-XRD) and thermogravimetry with differential thermal analysis (TG-DTA) were carried out. As seen in VT-XRD, **1** was decomposed and the formation of TiO_2_ began over 400 °C (Fig. 4a). This result of VT-XRD was well matched with that of TG-DTA. TG-DTA showed the weight loss over 250 °C because of evaporation of acetonitrile. Note that the exothermic peak was observed in DTA over 350 °C (Fig. 4b). The exothermic peak is ascribed to the oxidation of titanium coordination clusters to form TiO_2_. The results of VT-XRD and TG-DTA suggested that TiO_2_ began to be crystallized over 350–400 °C.

Quadrupol mass spectroscopy (Q-MS) of **1** under heating further gave the insight into gas generation and mechanism of nitrogen doping. As seen in Fig. 4c, the gases of benzene, aniline, HNO_3_ and CO_2_ were generated in the temperature region of 300–480 °C, suggesting the decomposition of 4-aminobenzoate. The organic ligand was decomposed to generate gases concurrently with the formation of TiO_2_. In other words, TiO_2_ was crystalized during the generation of gases.

The decomposition of 4-aminobenzoate into benzene indicates that the covalent bond between the amino-group and phenyl ring was cleaved to generate the fragments containing nitrogen atoms (N-fragment). The generation of N-fragment was also confirmed by the detection of HNO_3_. HNO_3_ was most likely formed by the oxidation of N-fragments. The rest of N-fragments reacted with TiO_2_ and nitrogen atoms were incorporated into TiO_2_ as a dopant.

This synchronic reaction was also observed in **2**. **2** was decomposed to begin the formation of TiO_2_ over 400 °C, which was characterized by VT-XRD and DT-XRD (Figure S7 and Figure S8). Q-MS measurement of **2** showed that benzoate was decomposed into gases of benzene and CO_2_ in 300–480 °C. Gas generation and formation of TiO_2_ were overlapped in the temperature range of 350–480 °C (Figure S9). Nitrogen was not doped into TiO_2_-(**2**) because of no nitrogen source (amino group) in the starting material of **2**. However, gas generation during the formation of TiO_2_ also resulted in the formation of mesoporous TiO_2_ (Figure S10).

**Figure 5:**
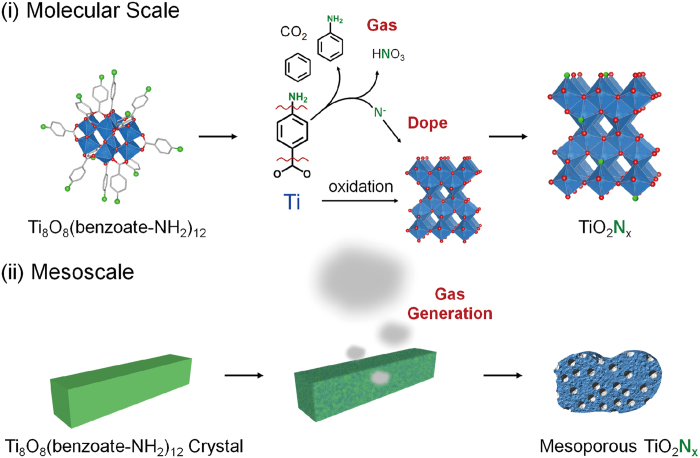
Schematic illustration of the reaction mechanism. Reaction scheme at (**i**) molecular scale and (**ii**) mesoscale: Coordination cluster of **1** is converted to mesoporous N-doped TiO_2_.

Based on VT-XRD, TG-DTA, and Q-MS, we propose following the reaction mechanism of nitrogen doping. Ti_8_O_10_(4-aminobenzoate)_12_ was decomposed to form TiO_2_ over 350 °C. 4-aminobenzoate of **1** was decomposed into the gases of aniline, benzene, CO_2_ and N-fragments. Nitrogen atoms in N-fragments reacted with TiO_2_ to be incorporated into TiO_2_ as a dopant, forming N-doped TiO_2_ (Fig. 5(i) molecular scale). The gases, including CO_2_, benzene, were generated concurrently with the formation of TiO_2_. Thus, gas evaporation during the formation of TiO_2_ created internal voids, leading to the formation of mesoporous N-doped TiO_2_ (Fig. 5(ii) mesoscale). As mentioned above, the surface area of TiO_2_-(**1**) and TiO_2_-(**2**) are larger than the metal oxides synthesized by calcination of extended coordination frameworks^50^. This is because the gas generation synchronized with formation of TiO_2_ created mesopores and significantly increased the surface area.

To evaluate the advantage of the new synthetic method, we synthesized mesoporous N-doped TiO_2_ by a sol-gel method as a reference (TiO_2_-sg)^40^,^41^. Isopropanol solution of titanium isopropoxide was mixed with aqueous solution of urea and nitric acid to prepare precursor sol. The resulting sol was calcined to synthesize mesoporous N-doped TiO_2_. The nitrogen originating from urea was doped into TiO_2_. The mesoporosity and BET surface were evaluated by N_2_ adsorption (Figure S10). The mesoporosity is attributed to the interparticle voids as described in previous literatures^41^. As shown in Table S1, The BET surfaces of TiO_2_-(**1**) and TiO_2_-(**2**) were more than twice as large as that of TiO_2_-sg (TiO_2_-(**1**): 170.6 m^2^/g, TiO_2_-(**2**): 139.8 m^2^/g, TiO_2_-sg: 59.24 m^2^/g). The crystallinity of TiO_2_-(**1**) and TiO_2_-(**2**) is nearly same as TiO_2_-sg (crystallite size; TiO_2_-(**1**): 13.4 nm, TiO_2_-(**2**): 15.8 nm, TiO_2_-sg: 16.4 nm) (Figure S11 and Table S2). However, the concentration of nitrogen in TiO_2_-sg was slightly higher than TiO_2_-(**1**) (Figure S12-13).

**Figure 6:**
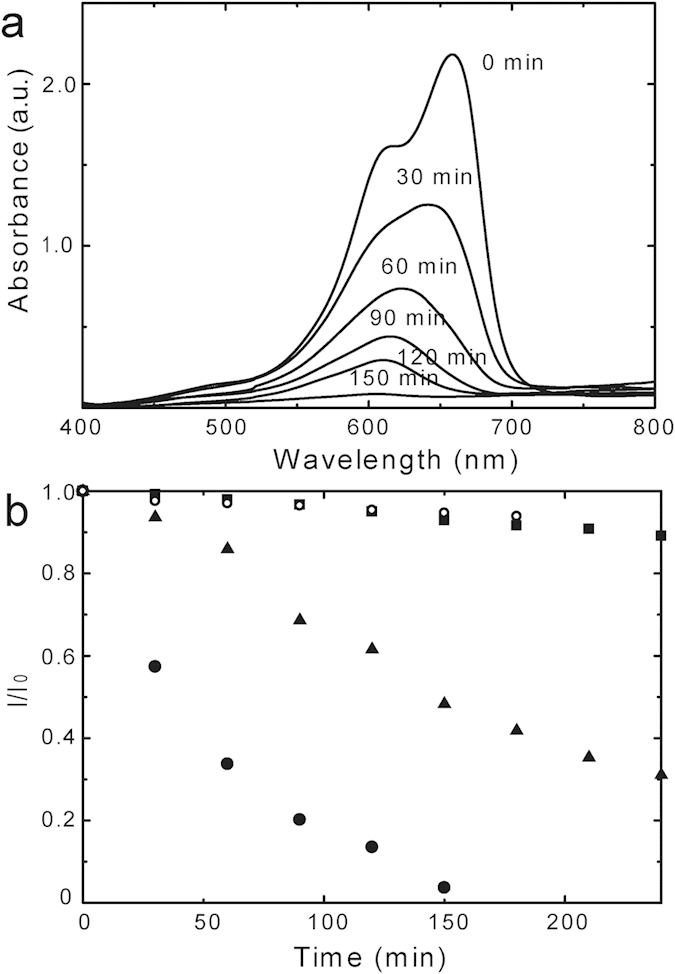
Photocatalytic activity of N-doped TiO_2_. (**a**) UV-visible spectroscopic changes of methylene blue solution over TiO_2_-(**1**), (**b**) chronological change of adoption intensity upon various photocatalysts under visible-light (>410 nm) irradiation: no catalyst (circle), TiO_2_-(**1**) (black dot), TiO_2_-(**2**) (black square) and TiO_2_-sg (black triangle).

We evaluated the visible-light photocatalytic activity of TiO_2_-(**1**), TiO_2_-(**2**) and TiO_2_-sg by degradation of methylene blue (MB)^51^,^52^. The crystals of TiO_2_-(**1**), TiO_2_-(**2**) or TiO_2_-sg were placed in a solution of MB and vigorously stirred under visible-light irradiation (> 410 nm). The absorption intensity of MB decreased over time, showing the photocatalytic activity of TiO_2_-(**1**) and TiO_2_-sg for the degradation of MB (Fig. 6 and Figure S14). The decrease rate of TiO_2_-(**2**) and no catalysts were nearly same, suggesting that the intensity decrease of MB was attributed to not the adsorption of MB on TiO_2_ particles but the photocatalytic decomposition of MB. Although the nitrogen concentration of TiO_2_-sg was higher than TiO_2_-(**1**), TiO_2_-(**1**) decomposed MB much faster than TiO_2_-sg. MB was completely decomposed by TiO_2_-(**1**) in 150 min, while only a half amount of MB was decomposed by TiO_2_-sg. Since nitrogen concentration of TiO_2_-(**1**) is lower than TiO_2_-sg, the rapid degradation of MB is most likely attributed to the large surface area of TiO_2_-(**1**). The mesoporous N-doped TiO_2_ can be easily prepared by calcination of the coordination cluster, but shows better photocatalytic activity than the one synthesized by a conventional sol-gel method.

## Conclusion

In this contribution, we demonstrate a facile method for the synthesis of mesoporous anion-doped metal oxides. As a model system, we synthesized a multinuclear titanium coordination cluster with a pendant amino-group. A simple calcination of the coordination cluster resulted in synchronic reactions: thermal oxidation of the coordination cluster into TiO_2_ and gas generation including N-fragments. The gas generation during the formation of TiO_2_ allows the introduction of mesopores. Furthermore, nitrogen atoms in N-fragments reacted with TiO_2_ to be incorporated as nitrogen dopant, thus leading to the formation of mesoporous N-doped TiO_2_. The resulting mesoporous N-doped TiO_2_ showed photocatalytic activity under visible light better than TiO_2_ prepared by a conventional sol-gel method, because of its larger surface area.

Notably, coordination clusters can be rationally designed by a choice of metal ions and organic ligands. Besides, doping amount can be potentially controlled by optimizing calcination conditions of coordination clusters (Figure S15). The synthetic and calcination protocols of the coordination clusters do not require specialized instruments. Therefore, coordination clusters as precursors will be a promising method for anion-doped porous metal oxides, which will offer significant benefits for the fabrication of light emitting diodes, ion storage batteries and heterogeneous catalysts.

## Methods

### Synthesis of Ti_8_O_10_(4-aminobenzoate)_12_

A mixture of titanium(IV) isopropoxide (5.1 × 10^−2^ mL, 1.72 × 10^−1^ mmol) and benzoic acid (284 mg, 2.33 mmol) was suspended in acetonitrile (3 mL) and heated in a teflon-lined steel bomb at 100 °C for 1 day. The resulting crystals of Ti_8_O_10_(4-amino benzoate)_12_ (**1**) were harvested by centrifuge and washed with acetonitrile three times.

### Synthesis of Ti_8_O_8_(benzoate)_16_

A mixture of titanium(IV) isopropoxide (2.55 × 10^−2^ mL, 0.86 × 10^−1^ mmol) and benzoic acid (142 mg, 1.66 mmol) was suspended in acetonitrile (3 mL) and heated in a teflon-lined steel bomb at 100 °C for 1 day. The resulting crystals of Ti_8_O_8_(benzoate)_16_ (**2**) were harvested by centrifuge and washed with acetonitrile three times.

### Calcination of 1 and 2

Crystals of **1** or **2** are placed in an Al_2_O_3_ boat (Sansho, SAB-995). The crystals are heated up to 480 °C and kept at the temperature for 3 hours.

### Synthesis of N-doped TiO_2_ by Sol-Gel Method

N-doped TiO_2_ was prepare by a reported protocol with slight modifications^40^,^41^. Titanium(IV) isopropoxide (5.94 × 10^−1^ mL, 2.0 mmol) was added to 10 ml of isopropanol. Subsequently, urea (120 mg, 2.0 mmol) and nitric acid (25 μl) were mixed with deionized water (0.36 mL). The solution of urea was dropped into the solution of titanium(IV) isopropoxide under stirring. The resulting sol was dried at 70 °C and calcined at 400 °C in air for 4 hours.

### Photocatalytic Activity Test

TiO_2_ (3 mg) was added to a quartz cell with 3 ml of MB solution (20 ppm). A halogen lamp (SX-UI502M, USHIO SPAX INC.) was used as the light source. 400 nm cut-off filter was placed in front of the reactor.

### X-ray Photon Spectroscopy (XPS)

Dried powders of TiO_2_-(**1**) and TiO_2_-(**2**) were placed on a carbon conductive tape to avoid the powders from swirling in the air. XPS data were collected by JEOL Ltd. JPS-9200.

### N_2_ Gas Adsorption

N_2_ adsorption measurements were carried out by Quantachrome Autosorb 6AG. The BET surface area was determined by the multipoint BET method using the adsorption branch in the relative pressure (*P*/*P*_0_) range of 0.05–0.3. The pore-size distribution was estimated by applying Barret, Joyner, and Halender (BJH) method to the desorption branch of the isotherms.

### Powder X-ray Diffraction (XRD)

PXRD data were collected by Bruker D8 Advance ECO. Scherrer equation is applied to 110 diffraction of anatase TiO_2_ to estimate the average size of crystallite for TiO_2_-(**1**), TiO_2_-(**2**) and TiO_2_-sg. The instrumental broadening estimated by a standard sample (Al_2_O_3_) is 0.042.

### Single Crystal X-ray Diffraction

Single-crystal XRD data collection (5° < 2*θ* < 55°) was conducted at 223 K on Rigaku ACR-7R diffractometer Mo-K*α* radiation (*λ* = 0.7105 Å) with Rigaku Mercury CCD system. The structures were solved by a direct method (SHELXS) and expanded using Fourier techniques. All calculations were performed using Yadokari-XG. Crystal data for **1**: C_44_H_24_N_7_O_17_Ti_4_, *monoclinic*, space group *P*21/*n* (no. 14), *a* = 12.430(5) Å, *b* = 24.443(9), *c* = 16.163(6) Å, *β* = 93.367(6), *V* = 4902.27 Å^3^, *Z* = 4, *T* = 223 K, *ρ*_calcd_ = 1.510 gcm^−3^, *μ*(Mo-Kα) = 0.706 cm^−1^; *R*_1_ = 0.0957, w*R*_2_ = 0.1812, GOF = 1.055. The hydrogen are severely disorder. (CCDC: 1406003).

### Quadrupole Mass Spectrometer (Q-MS)

The mass spectra of gases were collected by ULVAC APS-001 under heating of titanium coordination clusters (**1**) and (**2**).

### Other Apparatus

SEM images were collected by Phenom ProX. UV-vis absorption was measured by JASCO V-570. TEM image was collected by JEM-2100.

## Additional Information

**How to cite this article**: Hirai, K. *et al.* Gas-generated thermal oxidation of a coordination cluster for an anion-doped mesoporous metal oxide. *Sci. Rep.* **5**, 18468; doi: 10.1038/srep18468 (2015).

## Supplementary Material

Supplementary Information

## Figures and Tables

**Figure 1:**
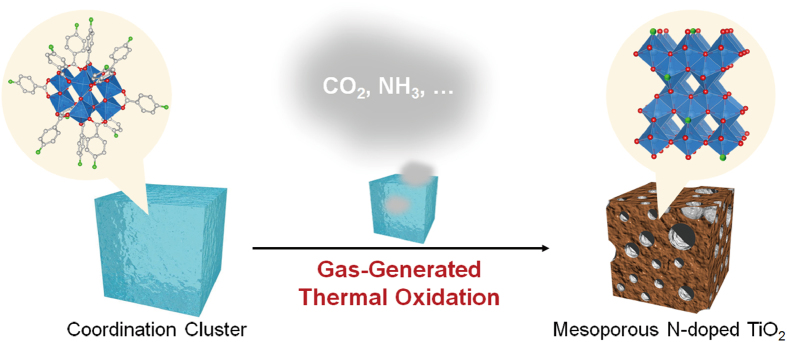
Schematic illustration of gas-generated thermal oxidation of a coordination cluster.
