# Supplementary material for: Gas-generated thermal oxidation of a coordination cluster for an anion-doped mesoporous metal oxide
Source: Sci Rep. 2015 Dec 18;5:18468. doi: 10.1038/srep18468 (PMC4683434; doi:10.1038/srep18468)
Supplement: Supplementary Information [file srep18468-s1.doc]

Supplementary Information

**Table of Contents**

SEM images of titanium coordination clusters before and after calcination 2

XPS spectra of TiO_2_-(**1**) and TiO_2_-(**2**) 3

XPS peak of C_1s_ 4

Raman spectra of TiO_2_-(**1**) and TiO_2_-(**2**) 5

N_2_ adsorption of TiO_2_-(**1**) and TiO_2_-(**2**) 6

TEM image of TiO_2_-(**1**) 7

VT-XRD of **2** 8

TG-DTA of **2** 9

Q-MS of **2** 10

N_2_ adsorption of TiO_2_-sg 11

BET surface areas of TiO_2_-(**1**), TiO_2_-(**2**) and TiO_2_-sg 12

XRD of TiO_2_-(**1**), TiO_2_-(**2**) and TiO_2_-sg 13

Crystallite estimated by Scherrer equation 14

XPS of TiO_2_-sg 15

UV-vis absorption of TiO_2_-sg 16

Chronological change of absorption intensity of MB 　 17

Calcination of **1** at slow heating rate 18

**SEM images of titanium coordination clusters before and after calcination**


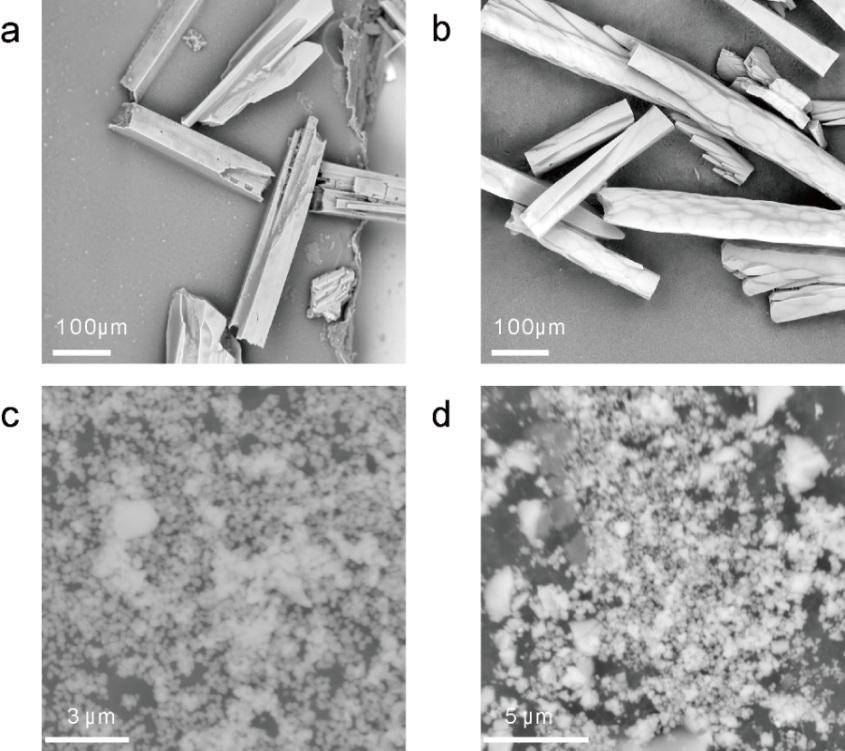


**Figure S1**. SEM images of (a) **1**, (b) **2**, (c) TiO_2_-(**1**) and (d) TiO_2_-(**2**)

**XPS spectra of TiO_2_-(1) and TiO_2_-(2)**

**
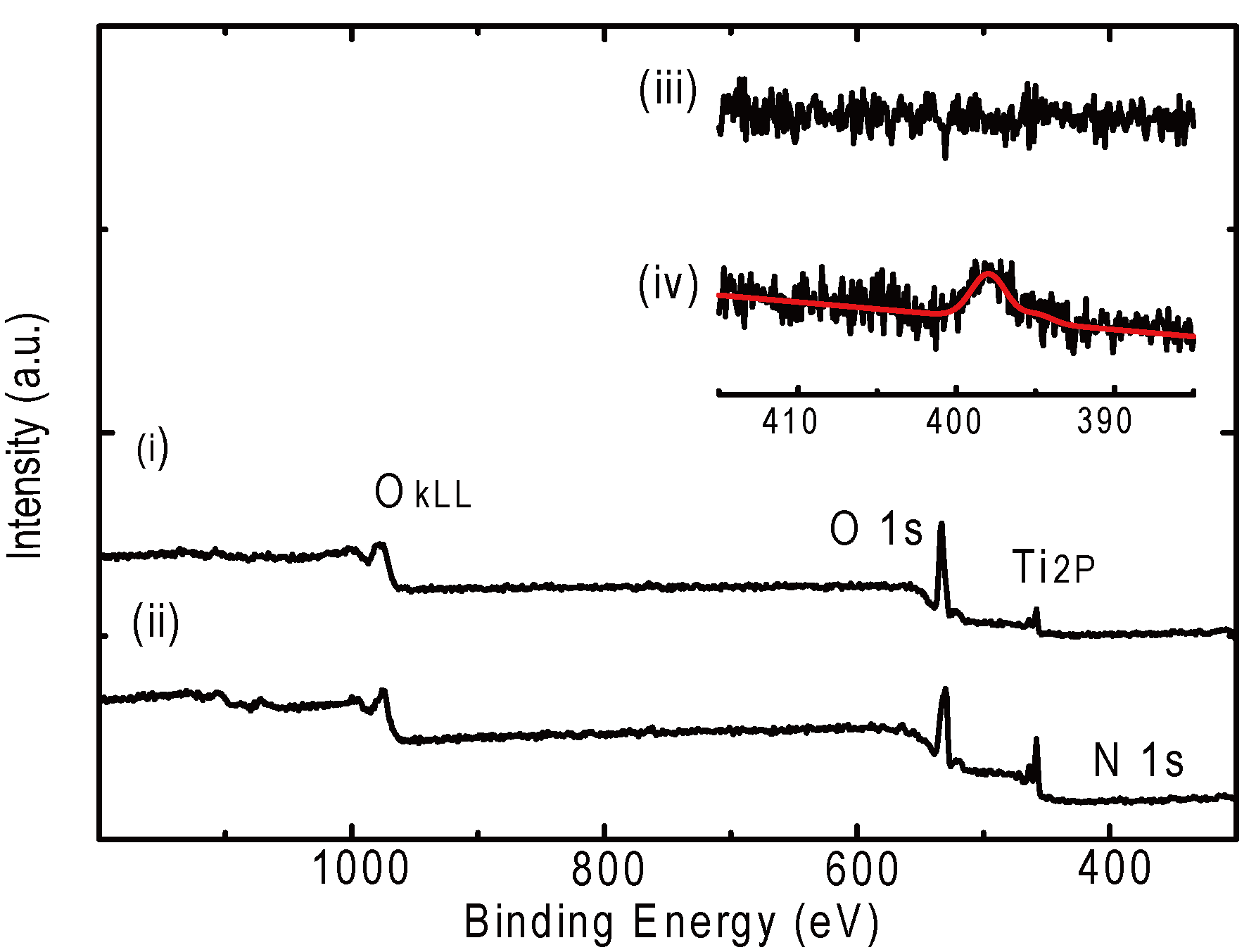
**

**Figure S2**. XPS spectra of (i) TiO_2_-(**2**) and (ii) TiO_2_-(**1**). Close-up of N_1s_ region: (iii) TiO_2_-(**2**) and (iv) TiO_2_-(**1**) with fitting curves (red).

**XPS peak of C_1s_**


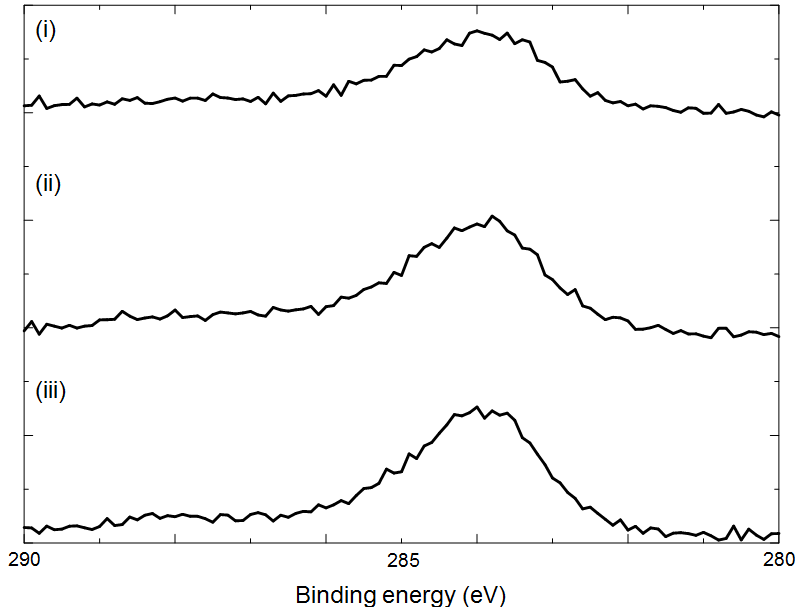


**Figure S3**. XPS peak of C1s for (i) TiO_2_-(**1**), (ii) TiO_2_-(**2**) and (iii) blank.

**Raman spectra of TiO_2_-(1) and TiO_2_-(2)**


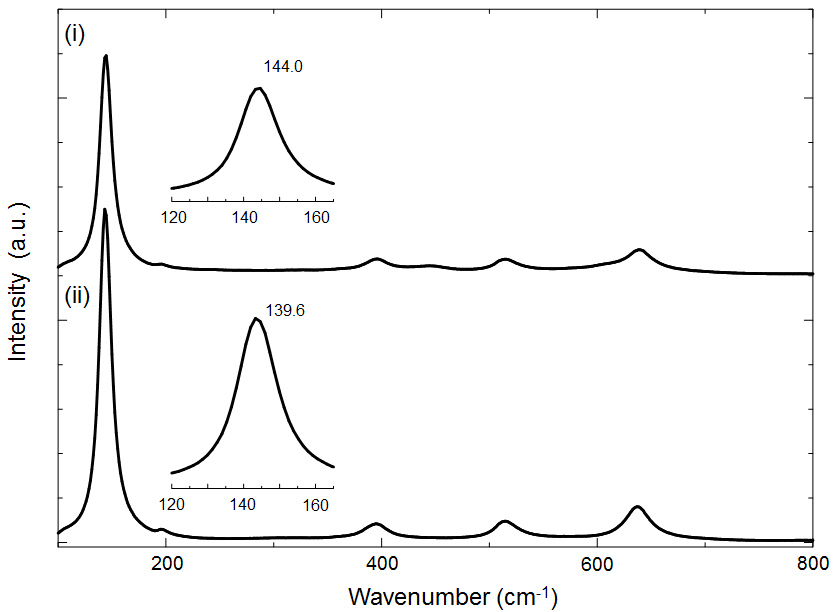


**Figure S4**. Raman spectra of (i) TiO_2_-(**1**) and (ii) TiO_2_-(**2**). The inset shows the close up of E_g(1)_ band. The inset shows the peak top of E_g(1)_ bands for TiO_2_-(**1**) and (ii) TiO_2_-(**2**) (144.0 and 139.6 cm^-1^).

**N_2_ adsorption of TiO_2_-(1) and TiO_2_-(2)**


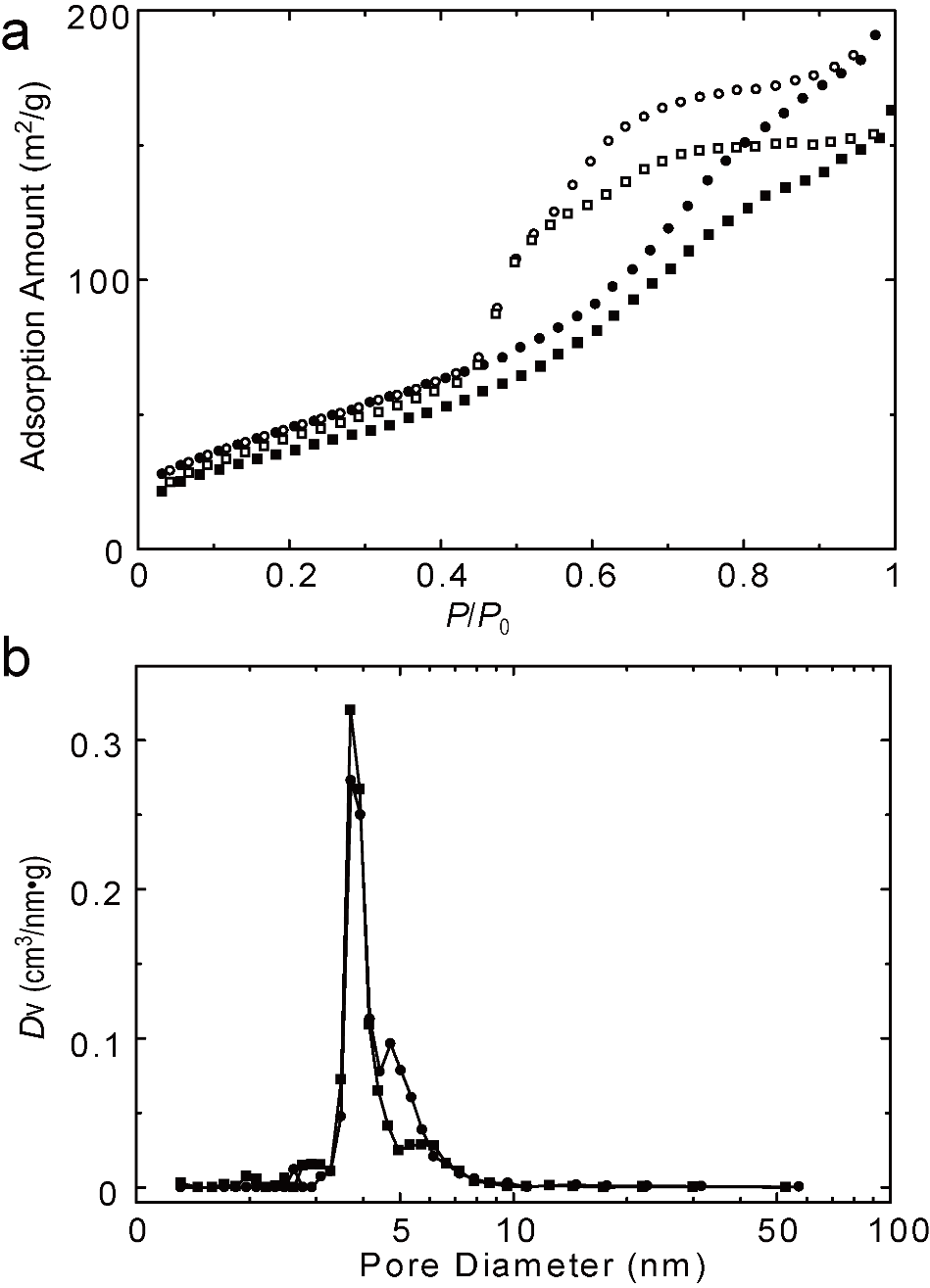


Figure S5. (a) N_2_ adsorption isotherms of TiO_2_-(1) (circle) and TiO_2_-(2) (square). Solid and open symbols indicate adsorption and desorption, respectively. (b) pore size distribution of TiO_2_-(1) (circle) and TiO_2_-(2) (square).

**TEM images of TiO_2_-(1)**

**
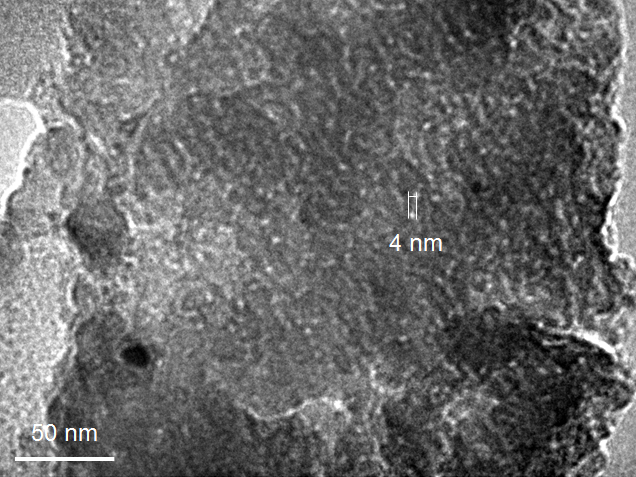
**

**Figure S6**. TEM image of TiO_2_-(**1**). The mesopores (white dots) were observed in TiO_2_ particles.

**VT-XRD of 2**


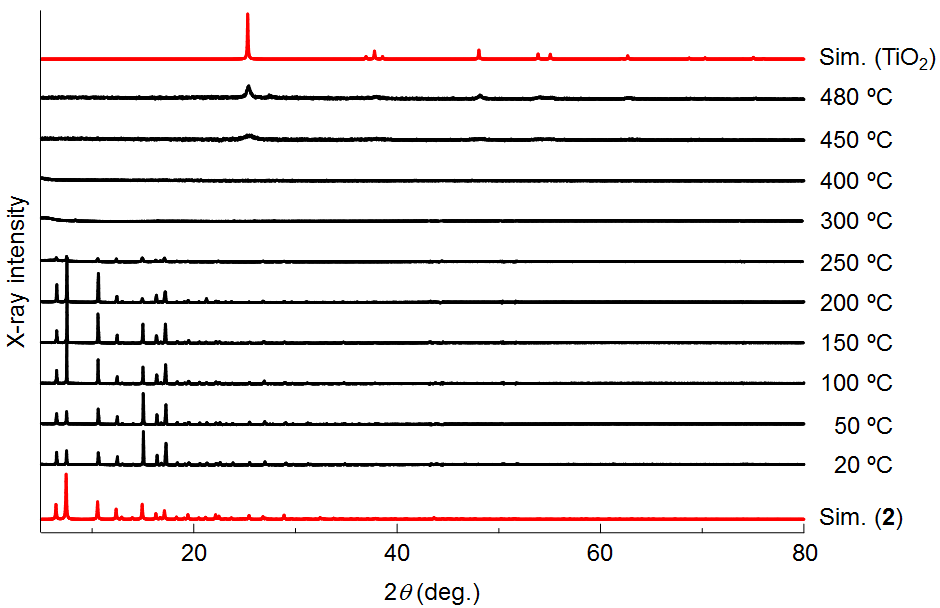


**Figure S7.** PXRD of **2** at variable temperatures from 20 to 480 °C.

**TG-DTA of 2**


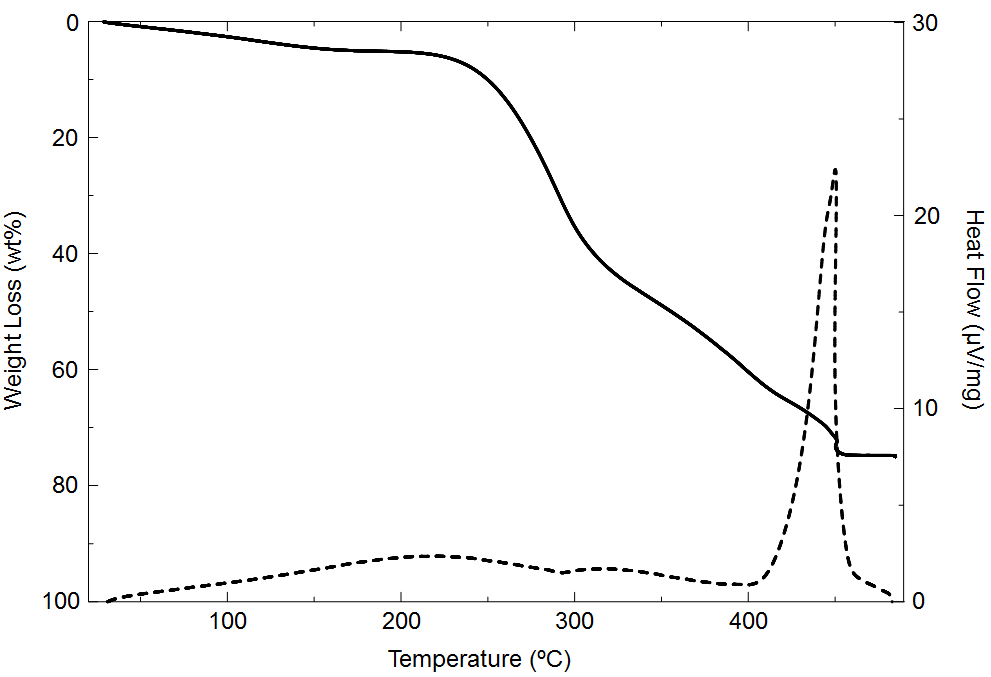


**Figure S8**. TG showing weight loss of **2** upon heating (black solid). DTA shows exothermal peak upon heating (black dots line).

**Q-MS of 2**


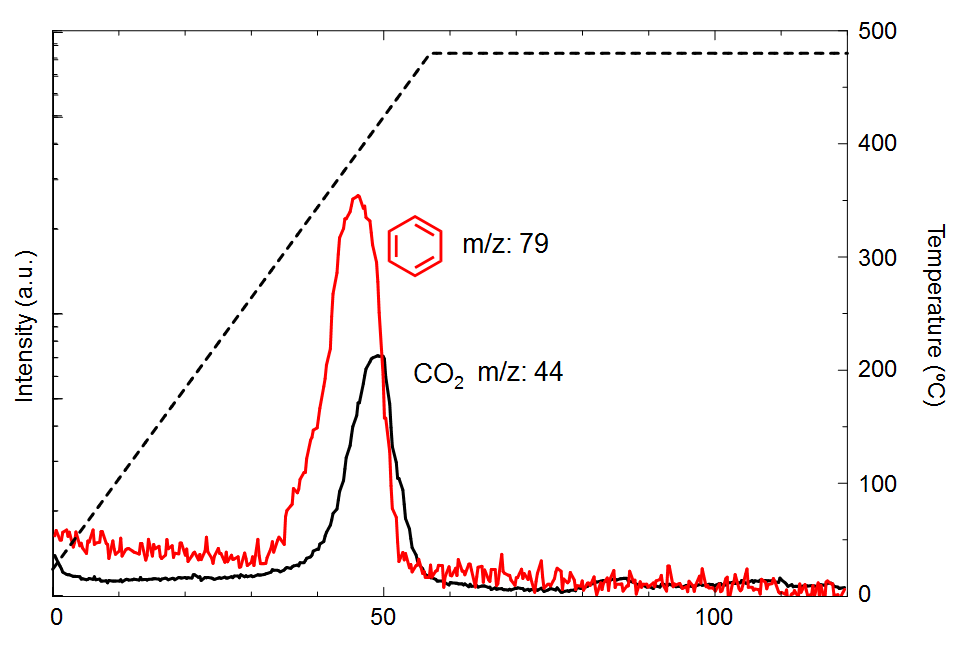


**Figure S9**. Q-MS analysis upon heating: benzene (red) and CO_2_ (black) were observed. Black dot line shows temperature of the sample cell.

**N_2_ adsorption of TiO_2_-sg**


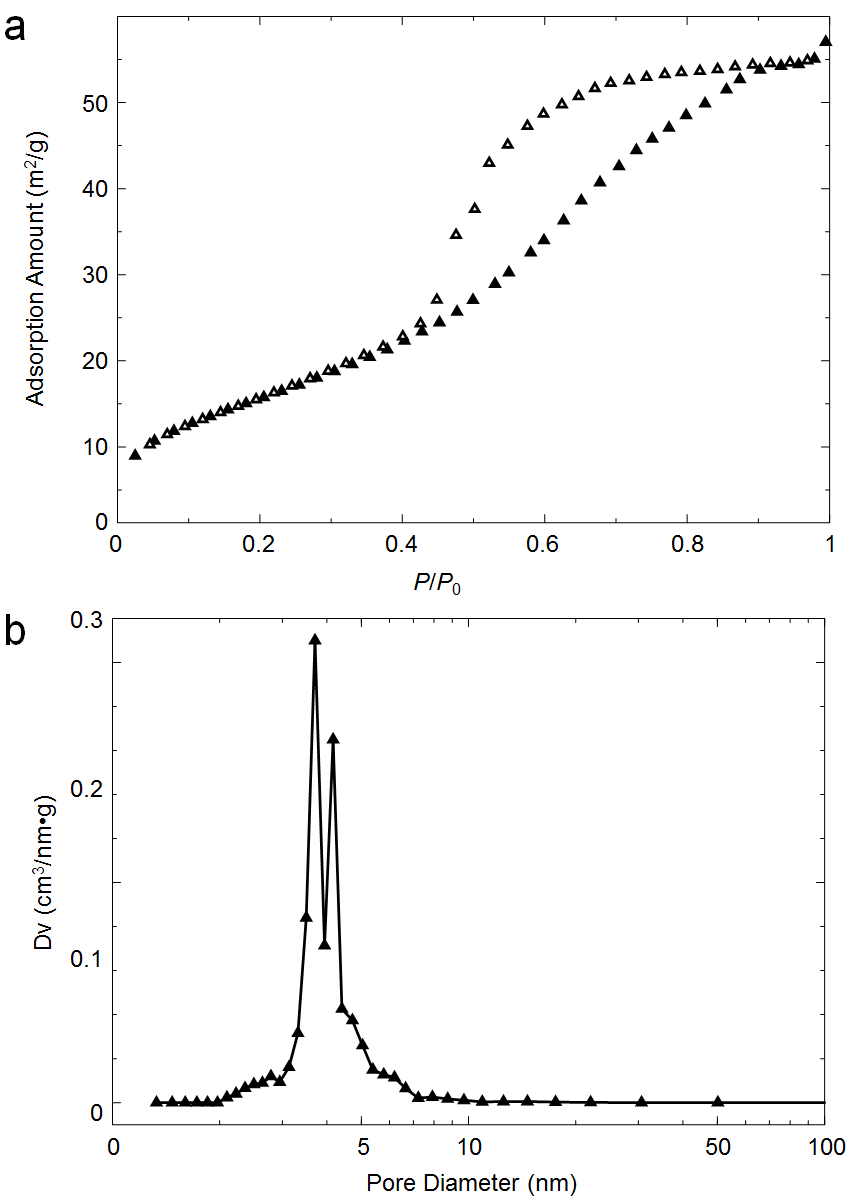


**Figure S10**. (a) N_2_ adsorption isotherms of TiO_2_-sg. Solid and open symbols indicate adsorption and desorption, respectively. (b) pore size distribution of TiO_2_-sg.

**BET surface area of TiO_2_-(1), TiO_2_-(2) and TiO_2_-sg**

**Table S1**. BET Surface and pore volume of TiO_2_-(**1**), TiO_2_-(**2**) and TiO_2_-sg estimated by N_2_ adsorption.

| Catalysts | BET Surface Area (m^2^/g) | Total Pore Volume (cm^3^/g) |
| --- | --- | --- |
| TiO_2_-(**1**) | 170.6 | 2.951 × 10^-1^  (*P*/*P*_0_ = 0.974) |
| TiO_2_-(**2**) | 139.8 | 2.519 × 10^-1^  (*P*/*P*_0_ = 0.996) |
| TiO_2_-sg | 59.24 | 8.822 × 10^-2^  (*P*/*P*_0_ = 0.995) |

BET surface were calculated by the uptake amounts in the region of *P*/*P*_0_ = 0.05 - 0.3.

**XRD of TiO_2_-(1), TiO_2_-(2) and TiO_2_-sg**

**
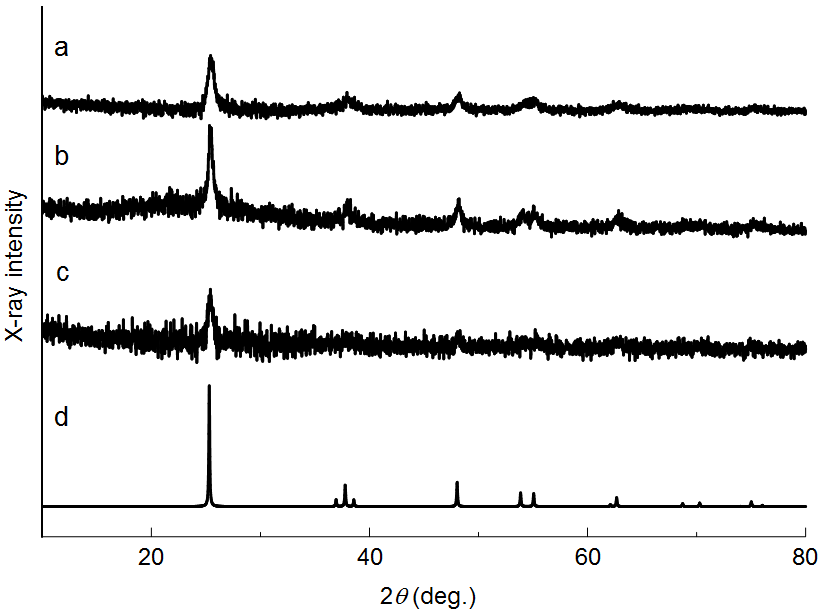
**

**Figure S11.** PXRD patterns of (a) TiO_2_-(**1**), (b) TiO_2_-(**2**), (c) TiO_2_-sg, and (d) simulated TiO_2_.

**Crystallite estimated by Scherrer equation**

Scherrer equation (eq-1) is applied to 101 diffraction of anatase TiO_2_ to estimate the average size of crystallite for TiO_2_-(**1**), TiO_2_-(**2**) and TiO_2_-sg. The instrumental broadening estimated by a standard sample (Al_2_O_3_) is 0.042.


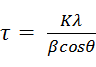
 - (eq-1)

*τ*: average size of crystallite

*K* (= 0.9): dimensionless shape factor

*λ* (= 1.5418): X-ray wavelength

*θ*: Bragg angle

*β*: peak broadening at half the maximum intensity

**Table S2. Average size of crystallite for TiO_2_-(1), TiO_2_-(2) and TiO_2_-sg.**

| Catalysts | *τ* (nm) |
| --- | --- |
| TiO_2_-(**1**) | 13.4 |
| TiO_2_-(**2**) | 15.8 |
| TiO_2_-sg | 16.4 |

**XPS of TiO_2_-sg**

**
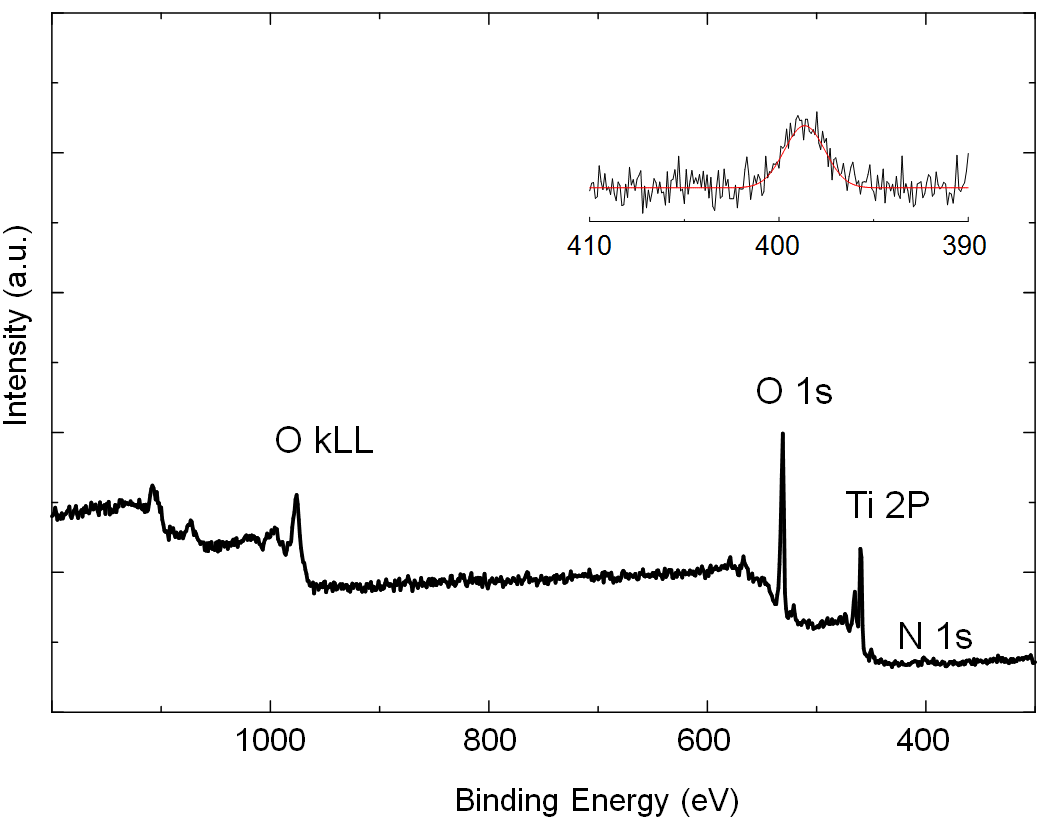
**

**Figure S12.** XPS spectrum of TiO_2_-sg. The inset shows a close-up of N1s region. The nitrogen concentration in TiO_2_-sg was estimated as 1.85 %

**UV-vis absorption of N-doped TiO_2_-sg**

**
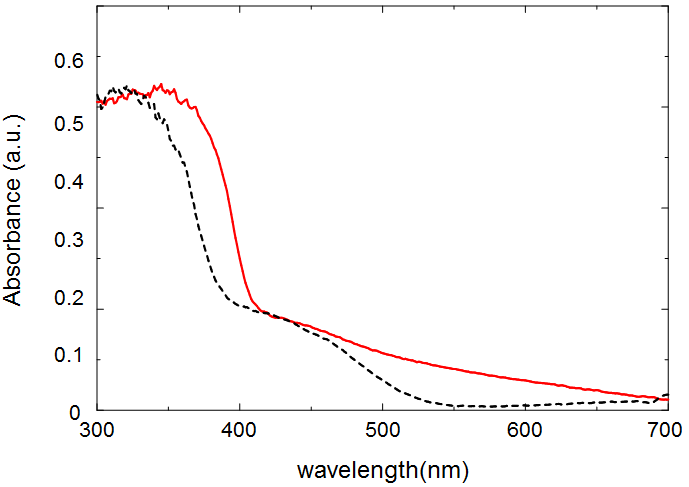
**

**Figure S13**. UV-vis absorption spectra of TiO_2_-(**1**) (red) and TiO_2_-sg (black dot line).

**Chronological change of absorption intensity of MB**

**
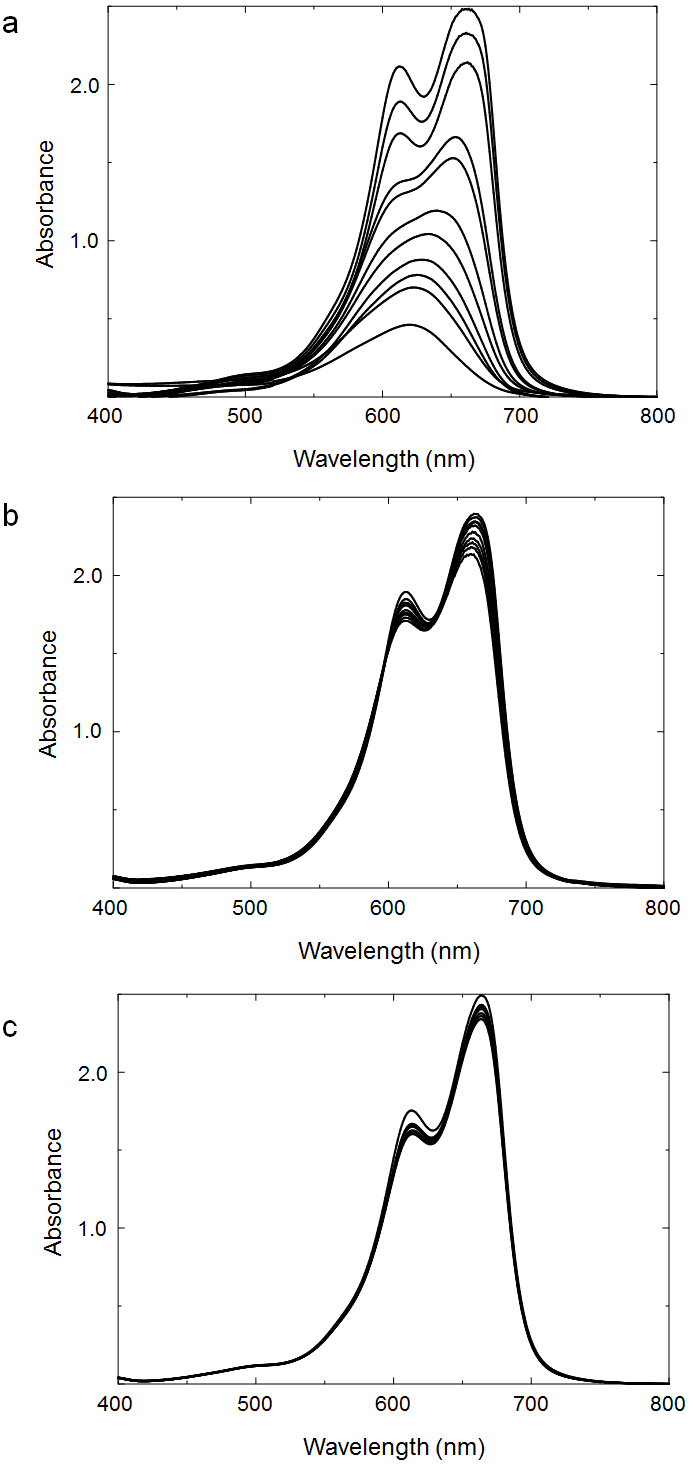
**

**Figure S14**. UV-visible spectroscopic changes of methylene blue solution over (a) TiO_2_-sg, (b) TiO_2_-(**2**) and (c) no catalyst.

**Calcination of 1 at slow heating rate.**

The coordination cluster of **1** was calcined with slow heating rate (3 °C/min). XPS spectra of resulting TiO_2_ showed the nitrogen concentration in TiO_2_ was less than 0.3 %, which is less than the nitrogen concentration in of TiO_2_-(**1**) (0.96 %). The nitrogen doping amount can be roughly controlled by optimizing the calcination concentration.

**
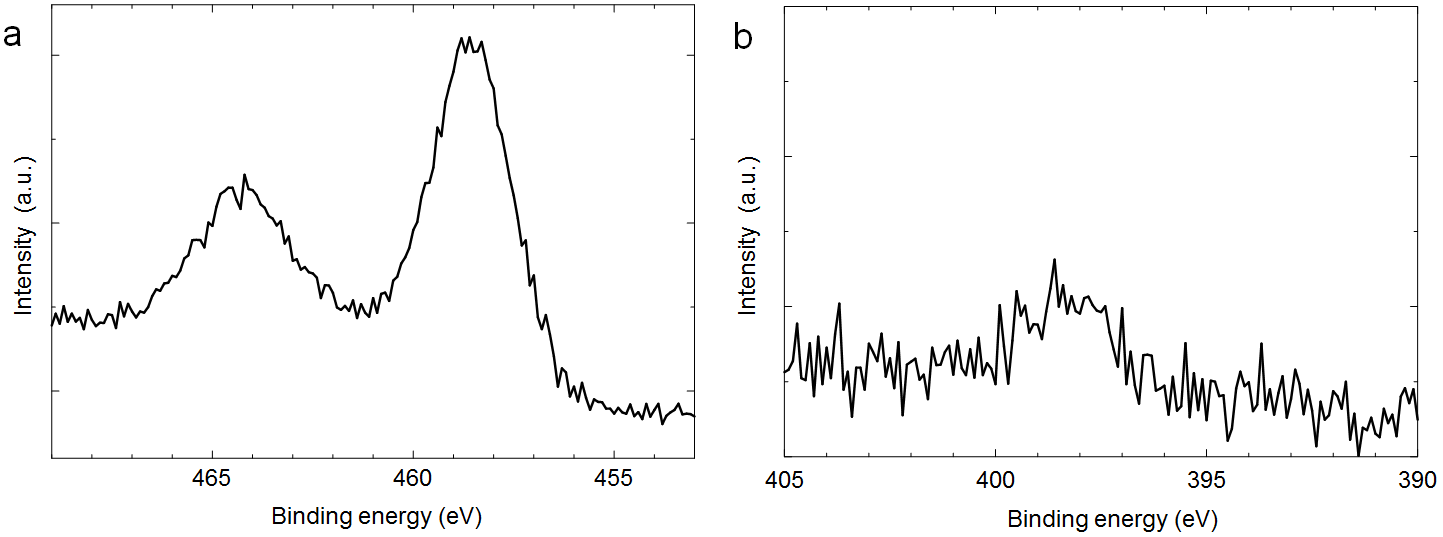
**

**Figure S15**. XPS spectra of TiO_2_-(**1**) for (a) Ti_2p_ and (b) N_1S_. The nitrogen concentration was calculated as less than 0.3 %.
